# Biomimicking Atherosclerotic Vessels: A Relevant and (Yet) Sub-Explored Topic

**DOI:** 10.3390/biomimetics9030135

**Published:** 2024-02-23

**Authors:** Joana Henriques, Ana M. Amaro, Ana P. Piedade

**Affiliations:** University of Coimbra, CEMMPRE, ARISE, Department of Mechanical Engineering, 3030-788 Coimbra, Portugal; joana.henriques@uc.pt (J.H.); ana.amaro@dem.uc.pt (A.M.A.)

**Keywords:** atherosclerosis, plaque types, multicomponents, biomimicking models, vascular models, biomechanics, hemodynamics, computational simulations, biomedical imaging, therapeutic validation, surgical planning, angioplasty

## Abstract

Atherosclerosis represents the etiologic source of several cardiovascular events, including myocardial infarction, cerebrovascular accidents, and peripheral artery disease, which remain the leading cause of mortality in the world. Numerous strategies are being delineated to revert the non-optimal projections of the World Health Organization, by both designing new diagnostic and therapeutic approaches or improving the interventional procedures performed by physicians. Deeply understanding the pathological process of atherosclerosis is, therefore, mandatory to accomplish improved results in these trials. Due to their availability, reproducibility, low expensiveness, and rapid production, biomimicking physical models are preferred over animal experimentation because they can overcome some limitations, mainly related to replicability and ethical issues. Their capability to represent any atherosclerotic stage and/or plaque type makes them valuable tools to investigate hemodynamical, pharmacodynamical, and biomechanical behaviors, as well as to optimize imaging systems and, thus, obtain meaningful prospects to improve the efficacy and effectiveness of treatment on a patient-specific basis. However, the broadness of possible applications in which these biomodels can be used is associated with a wide range of tissue-mimicking materials that are selected depending on the final purpose of the model and, consequently, prioritizing some materials’ properties over others. This review aims to summarize the progress in fabricating biomimicking atherosclerotic models, mainly focusing on using materials according to the intended application.

## 1. Introduction

**Figure 1:**
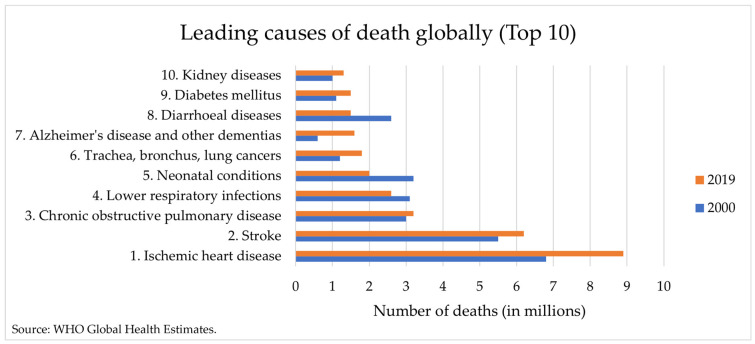
The top 10 causes of death globally in 2000 and 2019, presented by the World Health Organization (WHO). (Adapted from WHO [8]).

The World Health Organization (WHO) specifies cardiovascular diseases (CVDs) as the leading cause of death worldwide, with statistical projections predicting more than 23.6 million deaths in 2030 (Figure 1) [1,2]. These numbers have already triggered some apprehension in the scientific community, which foresees serious consequences at both health and socioeconomic levels if this epidemiologic problem is not solved promptly. Many efforts are being made to prevent such dangerous projections; for instance, WHO has even defined some targets to be achieved by 2025 regarding the halting of inherent factor risks, but these have been unsuccessful so far [2]. Therefore, it is mandatory to find alternatives to effectively reduce the CVD burden across the world, through increasing awareness campaigns and developing new treatment strategies. Moreover, improved knowledge about pathological conditions is also essential to establish correct therapeutic approaches.

**Figure 2:**
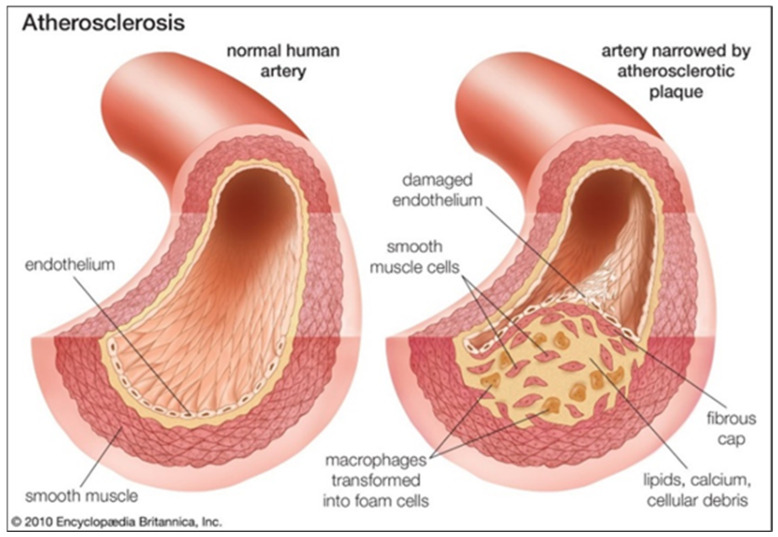
Comparison between a healthy and an atherosclerotic artery. The plaque formation in the inner surface of the artery leads to the narrowing (stenosis) of blood vessels and deficiencies of oxygen supply in tissues. The resulting plaques could comprise lipid cores, fibrotic areas, and/or calcifications. (Source: [9]).

The etiology of most of CVDs involves atherosclerosis [3]. Atherosclerotic disease is a chronic inflammatory condition, strongly associated with a significant remodeling of the arterial wall, both at structural and functional levels, and therefore is the cause of several deathly events, such as ischemic cerebral accidents and myocardial infarctions (Figure 2) [4,5,6]. Indeed, the most significant consequence of atherosclerosis is the development of multicomponent atherosclerotic plaques. These plaques not only lead to architectural arrangements affecting the biomechanical and physiological performances of the arterial wall but also cause the decrease in luminal areas. Consequently, there will be a reduction (or even obstruction) of the oxygen-containing blood flow downstream and, eventually, oxygen deficiencies in tissues and significant clinical emergencies [6,7].

As will be further discussed, atherosclerotic plaques are classified based on their development stage and composition. Generally, initial stages are difficult to identify, mainly because they are clinically asymptomatic. On the other hand, advanced stages are more concerning due to the vulnerability of plaque to rupture and severely block the artery, triggering serious clinical symptoms.

Accordingly, once detected, advanced-stage atherosclerotic disease must be immediately treated. Since it is instigated by multifactorial causes, single-intervention therapy may not be sufficient to reduce its incidence [10]. Modifying lifestyle risk factors, combined with pharmacotherapy, is the well-accepted primary preventive measure [2,11]. Surgical interventions, however, are imperative when a lethal event is imminent, i.e., when atherosclerotic plaques are severely occluding blood vessels (stenosis higher than 75%), and it is crucial to restore blood supply [3]. Several interventional approaches can be accomplished depending on the plaque composition and stage severity [12]: (1) balloon angioplasty, consisting of a small balloon transported via a catheter that is inflated inside the stenotic artery to reopen the blocked area and reestablish the blood flow [13]; (2) atherectomy, which involves direct removal of plaque build-up in the arteries, using a device placed at the end of a catheter that could use directional, orbital or rotational, excisional or aspiration, or laser technologies to remove the atherosclerotic plaque efficiently [14]; or (3) vascular stenting, which is the insertion of stents (hollow and tubular structures) in the obstructed artery to open the blocked area, prevent re-blocking of vascular lumen, and support the artery wall at the same time [13]. To ensure the success of these complex vascular interventions, patient-specific vascular biomodels should be designed for both surgical planning and procedural training.

Undeniably, creating physical biomodels capable of mimicking atherosclerotic blood vessels could be a tremendous asset to cardiology practices [15]. The produced biomodels could be used as experimental tools for the development and validation of new instruments, imaging systems, and protocols [16]. Moreover, they may enable personalized surgical planning and training [17,18,19] using vascular models generated from the imaging data sets of a particular patient (Personalized Medicine) [20].

Furthermore, these biomodels could serve as experimentation devices to study the biophysical and biomechanical mechanisms involved in atherosclerosis development and progression. For instance, through replicating an atherosclerotic condition, it is possible to infer how the shape and stiffness of plaques could affect the rheological properties of blood [21]. Consequently, as hemodynamics strongly influences atherosclerosis progression, a better understanding of the relationship between pathological conditions and mechanical performances will allow the designing of more effective pharmacological and interventional therapies. Numerical studies could also help in these studies, as they can evaluate the influence of atherosclerotic plaques on the hemodynamic parameters such as wall shear stress, relative residence time, and oscillatory shear index, commonly used in cardiovascular sciences [22]. However, these numerical studies must be validated to ensure that the results correspond to reality. This way, biomimicking models are, once again, valuable tools to accomplish the validation.

Additionally, biomodels could replace in vivo experiments, overcoming some related limitations (expensiveness, ethical issues, lack of reproducibility) [23]. Although 2D cellular cultures have already provided some insights about atherosclerotic pathophysiology [24], they cannot replicate the relevant microenvironments of the disorder. On the other hand, animal models are currently implemented to study the releasing profiles of drug-eluting stents, which, for instance, are related to the unpredictability of plaque size, composition, and location [25]. In this context, 3D biomodels are specified as better tools to represent the disease, considering its dimensionality and anatomical features [26].

Reliable models, however, need to use materials that accurately reflect the physical, optical, chemical, and mechanical properties of intended tissues to mimic the pathological conditions efficiently [26,27]. Furthermore, the choice of materials depends on the proposed application, with some properties prioritized over others.

Therefore, this review summarizes the existing research on the biomimicking materials selected to replicate both vasculature and plaque components. First, the atherosclerotic pathophysiology will be introduced to expose the several types of plaques and their multicomposition, which, in turn, must be fully recapitulated to obtain solid models. Since the envisioned goal dictates the most crucial properties to be addressed in a particular model, the diversity of possible materials is also extensive. Consequently, biomimicking materials used in the literature to fabricate biomodels, organized according to the application field, will be discussed. Due to the broadness of possible implementations, they are categorized into (1) imaging calibration and optimization, (2) flow and hemodynamic studies, (3) numerical simulations validation, (4) clinical surgical applications, and (5) recognition of novel treatment strategies.

## 2. Pathophysiology of Atherosclerosis

A deep understanding of the processes and mechanisms involved in atherosclerosis development and progression is imperative to represent in vivo pathophysiology effectively. Because atherosclerosis is a complex disease, it results from many biochemical phenomena leading to several atherosclerotic stages and, consequently, different plaque types (Table 1) [28].

Although the exact cause of atherosclerosis remains unknown, it is well-accepted that its pathogenesis relies on endothelial damage prompted by risk factors, including dyslipidemia, diabetes, and hypertension, accompanied by oxidative stress and an increased reactive oxygen species production [29]. Disturbed vascular endothelial function intensifies the vascular permeability to inflammatory cells from blood to endothelium and promotes the activation of pro- and anti-inflammatory signaling pathways [27]. These unbalanced events are supplemented by the secretion of cytokines and growth factors, resulting in the aggregation and activation of platelets and monocytes [30]. After being recruited to the intima, monocytes sequentially differentiate into macrophages and phagocytize oxidized low-density lipoproteins (LDL), forming foam cells [31]. Early atherosclerotic stages, considered reversible pathological changes [3], encompass these foam cells embedded in an extracellular matrix (ECM) with few inflammatory cells and fatty streaks. The progression of atherosclerosis is driven by the collagen density of ECM, as demonstrated by Garcia-Sabaté and colleagues [26].

Intimal disorganization is triggered, allowing the sedimentation and agglomeration of lipid-filled foam cells, forming atheroma, a lipid core isolated from the lumen via a surrounding proteoglycan-rich cap [28].

Meanwhile, vascular smooth muscle cells (VSMCs) are activated and suffer a phenotypic transformation, migrating from media to intima and progressively thickening arterial walls. Hyperproliferative VSMCs lead to intimal hyperplasia and, together with the overproduction of matrix metalloproteinase via macrophages that degrades interstitial collagen, a fibrous plaque is formed [3]. Incorrect differentiation of VSMCs into osteoblasts occurs, and a mineralized matrix is generated in a process analogous to the bone formation mechanism. Deposition of calcium salts leads to calcified plaques, related to advanced stages of atherosclerosis, usually causing stenosis [32].

As atherosclerosis evolves, a large number of foam cells undergo apoptosis and necrosis and consequently collapse, releasing lipids. The final atherosclerotic stage is, thus, a fibrotic lesion characterized by a fibrous cap and a necrotic core comprising lymphocytes, collagen, cholesterol crystals, lipid debris, and necrotic cells [3].

Hence, it is crucial to know the elements composing each plaque type to accomplish reliable results regardless of the envisioned application [33]. Concerning atherosclerosis diagnosis, the composition of atherosclerotic plaques has more value than the severity of lumen stenosis when it is necessary to predict plaque rupture, thrombosis, and ischemic events [28,34]. This proves the relevance of producing models that accurately biomimic the components of atherosclerotic plaques.

On the other hand, atherosclerosis development leads to an architectural rearrangement of arterial walls, interfering with the biomechanical properties of those blood vessels. Therefore, a reliable biomimicking model will demand a consciousness on the mechanical properties of both vessel wall and plaque components. Regarding this topic, the reader should carefully explore previous work [27], where the possible tests used to mechanically characterize atherosclerotic biological tissues, as well as the dispersibility of results, were addressed. Indeed, the variability among subjects, animal models (swine, mouse, ovine, etc.), artery typologies (femoral, coronary, carotid), and atherosclerotic stages is the source of the non-consensual results.

## 3. Applications of Biomimetic Vascular Models for Atherosclerosis

Due to atherosclerotic fatality, there is a demand for promptness in reverting the pessimistic WHO projections, which translates into the quite dispersed action lines from pharmacotherapy to novel surgical interventions. Despite the success of the existing strategies, they can be limited in terms of applicability because they are commonly designed for a specific group of patients and plaque types. A further step will include the management of any patient-specific vascular anatomy and condition. Therefore, it is indispensable to accomplish readily available, well-controlled, and low-priced experimental models for atherosclerotic blood vessels with predictable and reproducible plaque composition and location [25]. The potentiality and significance of these engineered biomodels will substantially impact CVD research.

To address these urgent needs, the capabilities of biomodels could extend from studying the hemodynamic and biomechanical mechanisms of atherosclerosis development to improving image-based diagnostic techniques and designing more effective therapies. Hence, the design and fabrication of any of these experimental models must consider the properties of natural human diseased vessels [22]. However, the most relevant properties to be mimicked are distinct for biomodel purposes.

### 3.1. Imaging Protocols Optimization

Due to the dangerous consequences of atherosclerosis, its diagnosis is tremendously relevant. Clinical assessment of atherosclerosis is usually performed with imaging techniques, such as computed tomography angiography (CTA), magnetic resonance angiography (MRA), and ultrasonography, in which Doppler ultrasound (US) is included [35]. Technological advances improved imaging protocols used in these diagnostic techniques. However, validating and optimizing such findings requires physical testing tools capable of simulating different parts of the human systems [18]. This can be accomplished using biomimetic models commonly designated as phantoms.

As stated by Stupic et al. (2021) [36], an imaging phantom is an “inanimate object used to characterize or calibrate imaging systems”, which must be accepted by the imaging community and connected to the International System of Measurements. Namely, phantoms should have stable and well-established properties to monitor imaging system performance and accuracy. However, the specific properties of phantom materials depend on the imaging system and technique under validation and optimization.

#### 3.1.1. Ultrasonography

When designing and constructing arterial phantoms to be used in ultrasonography, either to tutor ultrasonographers or evaluate the effectiveness of an ultrasound system, some specific properties must be considered. Namely, the mimicking materials must have density, speed of sound, viscosity, attenuation, and backscatter properties [37], similar to the internationally accepted standards by the International Electrotechnical Commission IEC 61685 [38].

For instance, Wong and colleagues [39] have investigated the performance of 5 polymers to be used as vessel-mimicking materials in the fabrication of Doppler US flow phantoms. Besides acoustic properties, authors considered the machinability, ease of construction, cost, and availability of materials. Candidate materials (Teflon^®^, polyurethane-90A, polyurethane-75D, high-density polyethylene, and ultra-high-molecular-weight polyethylene) were direct-machined into a geometry as prescribed by North American Symptomatic Carotid Endarterectomy (NASCET) criteria [40] and characterized by a pulse-transmission technique at a frequency of 5 MHz. Among tested materials, Teflon^®^ showed better values of speed of sound and attenuation, meaning that it was the most suitable material for phantom production, presenting the best combination of rigidity, reproducibility, and Doppler US compatibility [39].

**Figure 3:**
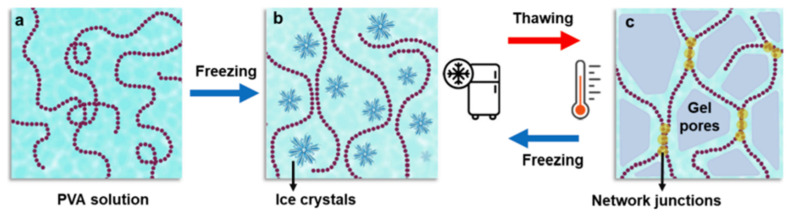
Schematic illustration of the preparation of PVA cryogels, via the freeze–thaw method. Firstly, PVA chains are dissolved in water (**a**); PVA chains became entrapped within ice crystals, due to phase separation from freezing step (**b**); and finally, ice crystals are transformed into pores of gel networks as the thawing step takes place (**c**). As the number of freeze–thaw cycles increases, the strength of gel also increases. (Source: [45], with permission).

Nevertheless, poly(vinyl alcohol) cryogel (PVA-C) is the most explored vessel-mimicking material to produce atherosclerotic phantoms in the optimization of US systems (Figure 3). Several studies report its utilization, with results proving its feasibility [22,35,41,42,43,44].

Galluzzo and co-workers [41] have designed carotid artery phantoms containing hypoechoic (or soft) and hyperechoic (or hard) plaques using PVA-C with tunable density through varying the number of freeze–thaw cycles. This study aimed to assess B-mode US imaging resolution in the measurement of the internal diameter of phantoms. Both vessel wall and plaques were obtained with PVA-C poured into molds; however, while hard plaques comprised PVA-C undergoing more cycles than the rest of the wall, soft plaques were produced via mixing butter and PVA-C undergoing a smaller number of cycles, assimilated to a lipid core plaque. Aluminum oxide powder was used to simulate acoustic scattering properties, and benzalkonium chloride was the antimicrobial agent. Accurate measurements of lumen diameters allowed one to conclude that these phantoms provided a realistic US acquisition setup to efficiently conduct US-based investigations, with the advantage of being very flexible regarding applicability, reproducibility, and economics [41].

Similarly, a study was conducted [42] to create PVA-C-based phantoms and validate US imaging systems. However, contrary to previously published work [41], diseased arteries were not carried out by inserting plaques inside the vessel phantom but rather by including an eccentric constriction in the 3D computer-aided design, following NASCET standards [40]. Acoustic scattering particles and antimicrobial agents were also different, with graphite microparticles and potassium sorbate used as scattering and antimicrobial agents, respectively. The resulting phantoms proved their acoustic and physical compatibility for ultrasound imaging experiments, including flow dynamics and wall motion [42].

With a similar purpose, researchers [43] have manufactured carotid artery phantoms containing atheromatous plaques inside to assist in optimizing flow imaging applications. Once again, PVA-C with varying freeze–thaw cycles was used to mimic the vessel wall (Young’s modulus of 342 ± 25 kPa) and the stenotic lipid pools (Young’s modulus of 17 ± 3 kPa); cellulose particles were implemented to improve the acoustic scatters. Mechanical testing, shear wave elastography, strain elastography, B-mode, color Doppler, and power Doppler images of produced phantoms were conducted to validate the fabrication process. Results indicated that phantoms were compatible with ultrasound imaging and reflected the shape and mechanical characteristics of normal and pathological stenosed carotid arteries. Future perspectives will include developing these phantoms using vascular and tissue rheology methods [43].

#### 3.1.2. Computed Tomography

Computed tomography (CT) and computed tomography angiography (CTA) belong to a long-established diagnostic technique capable of identifying atherosclerotic diseased vessels with luminal stenosis more significant than 50% based on the measurement of CT numbers [46]. Due to their higher contrast relative to surrounding tissues, calcified plaques are usually effortlessly detected. However, plaque vulnerability, and consequently acute myocardial risk, is strongly associated with plaque composition, which is as high as the non-calcified content [27]. Some studies report that CT numbers of lipid-rich and fibrous plaques range from −30 to 60 Hounsfield units (HU) and from 60 to 150 HU, respectively [47,48,49]. Nonetheless, these CT numbers depend on some parameters, including the measurement location within the wall relative to the center of the vessel and arterial diameter, tapering the measured values for larger arteries [50,51]. For this reason, optimizing CTA imaging protocols to characterize plaque composition effectively is a must.

For instance, to assess the influence of size, degree of stenosis, luminal contrast attenuation, and plaque geometry on stenosis quantification, a research group [50] has selected two materials to produce vascular phantoms with plaques based on their attenuation properties and their processability through Polyjet technology. To mimic the vessel wall and fibrous tissue, they used FullCure720 (transparent) with CT attenuation of 105 HU, and to replicate lipid plaques, they used FullCure920 Tango Black (flexible) with 72 HU of CT attenuation. An iodine–saline solution was introduced into tubular structures to simulate the contrast agent used in CTA. Although the measured degrees of stenosis from imaging were close to the produced stenosis, the rigidity of phantoms hampers their application in studies where vascular motion (due to the heart beating) must be implemented [50].

Analogously, another research group [52] fabricated vascular phantoms to evaluate the accuracy of lumen area measurement using a modified dual-energy CT protocol. They investigated the effects of contrast material concentration, vessel diameter (5.7, 4.9, 3.9, 3.0, 1.9, and 1.3 mm), and thickness of calcified plaque (2.0 vs. 4.0 mm) on this measurement. Phantoms were made of polyethylene tubes to mimic vascular tissue and calcium hydroxyapatite powder dissolved in liquid agar to simulate artificial hard calcified plaque. The results indicated that extensively calcified plaques lead to an underestimated measurement of the lumen area, meaning a false diagnosis of vascular stenosis; likewise, this underestimation was as low as the vessel diameter was large. Subsequently, through preventing in vivo experimentation, this study proved that further investigation must be conducted before implementing these protocols in clinical practice [52].

Furthermore, a dynamic physical phantom with a realistic coronary plaque was developed to investigate the accuracy of stenosis measurement using CT imaging techniques [51]. This study intended to evaluate the feasibility of using a retrospective gated coronary CTA protocol coupled with synthesized motion-synchronized electrocardiogram waveforms in cardiovascular assessment. Vascular models were 3D-printed using Polyjet technology. VeroWhite^®^ material was used to mimic the vessel wall, and the lumen was filled with iodinated contrast material. Calcified plaque consisting of a dental resin mixture was inserted into the 3D-printed void and cured with UV light; canola oil, in turn, was used to glue the plaque inside the cylinder and to biomimic the epicardia fat surrounding the coronary vessels. Phantoms were useful in determining the accuracy of CT number measurements and image quality under realistic acquisition conditions [51].

**Figure 4:**
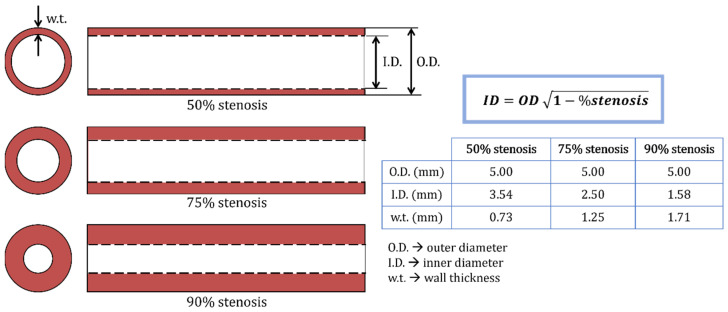
Dimensions of tubular samples prepared for CT scanning. Each sample was 3D-printed with the materials and technologies previously described in the text. [53] with permission.

More recently, plaque phantoms have been designed to investigate the effect of non-calcified plaque geometry and arterial motion on CT number measurements and consequently improve the ability of radiologists to characterize plaque type [53]. They studied four flexible 3D-printing materials and three additive manufacturing technologies: (i) Polyjet technology, using TangoGrey and TangoBlack resins; (ii) fused filament fabrication (FFF) technology using a polyurethane (TPU) filament; and (iii) stereolithography (SLA), in which a flexible resin was photopolymerized (from Formlabs Inc., Amsterdam, Netherlands). Tubular samples with varying stenosis degrees (Figure 4) were fabricated. An iodine contrast solution (200 HU) was used to fill each sample, which, in turn, was placed into a bolus material to simulate the chest wall and, thus, obtain a realistic acquisition setting. FFF was found to be unsuitable for producing mimicking phantoms due to its poor surface resolution, but SLA and Polyjet have fabricated well-resolved phantoms. The averaged CT numbers were below the accepted range for mimicking lipid-rigid plaques. The reason for these results was the similarity between the thickness of samples (0.73 mm to 1.71 mm) and the pixel size (0.5 mm) used in CTA imaging, which may have introduced some underestimations [53]. Although the feasibility of 3D printing to produce vascular biomimicking models was proven, some details must be improved, namely the tissue-mimicking materials selected.

Recently, Mørup and co-workers [54] tested four different materials (two gelatin mixtures, swine cardiac tissue from pigs, and Ecoflex^TM^ silicone) to determine the most adequate to simulate in vivo data regarding CT imaging, namely CT attenuation properties (in HU). They found that Ecoflex^TM^ had the closest attenuation value compared to real data, meaning that it is the most adequate to produce the phantoms utilized in optimizing CT protocols [54].

#### 3.1.3. Magnetic Resonance Imaging

Magnetic resonance imaging (MRI) is a non-invasive imaging technique extensively used to obtain three-dimensional detailed anatomic images, differentiating body components based on relaxation times that are characteristic of tissue types [36]. Due to the excellent soft tissue contrast, MRI allows detection and monitoring of human diseases, such as atherosclerosis. Therefore, tissue-mimicking materials should have chemical and physical stability over extended periods, adequate density and radiofrequency interaction, relaxation times, and conductivities in the biological range of interest to manufacture MRI vascular phantoms [55]. Resins, gels, silicones, and PVA-C hydrogels have been commonly used to mimic vessel walls in MRI phantoms [56,57,58,59].

For instance, researchers [57] have produced an atherosclerotic phantom containing plaque components, like a fibrous cap and lipid core, to validate the applicability of MRI in stroke risk assessment. Two phantom types were built, comprising PVA hydrogel to mimic arterial wall properties. While one had a fibrous cap made of agarose, carrageenan, sodium azide, and water, the other had a lipid core mimicked through a mixture of vegetable fat, carrageenan, and sodium azide. Quantitative evaluation (lumen diameter, structural composition, and contrast-to-noise ratio) of both models was conducted, the reliability of the manufacturing technique was established, and the possibility of using high-resolution MRI to distinguish plaque components was proven [57].

This work was further improved [60], and the materials used to mimic atherosclerotic plaques were slightly changed. Namely, the fibrous cap was simulated using a mixture of gadolinium chloride, agarose, carrageen, sodium azide, water, and sodium chloride, and the lipid core was mimicked using a mixture of sodium azide, carrageen, and milk. Milk was implemented instead of vegetable fat because the amount of lipid component is more controllably achieved [60]. Results were similar to previous work [57], but phantoms were additionally implemented to optimize image acquisition before using a high-resolution MRI in clinical studies. Although additional testing is needed, this proof-of-concept was a remarkable advance in MRI atherosclerotic phantom design.

MRI technique has also been used for qualitative and quantitative flow assessment by measuring blood flow velocity using phase-contrast (PC) MRI [56]. However, due to the lack of knowledge about the accuracy of PC-MRI measurements, its implementation in clinical practice has been retarded [61,62]. To address this issue, Gadda and collaborators [63] developed and validated an MRI-compatible hydrodynamic phantom to understand, test, and optimize PC-MRI measurements. As the relaxation time value is a crucial parameter to be considered in the design of biomimicking MRI models, the authors have selected a water solution of CuSO_4_ with a relaxation time value similar to those of human soft tissues at clinical magnetic field intensities. Likewise, blood had to be mimicked because of the importance of acquiring flow velocity profiles to validate PC-MRI measurements. Simulated fluid was produced by an external company and had the same magnetic properties as human blood. Some imaging errors were observed, suggesting the capability of the phantom to accurately detect error sources, which must be corrected and adjusted [63]. This phantom was a robust standardization tool to calibrate MRI systems, which is fundamental for implementing these systems in clinical practice.

#### 3.1.4. Optical Coherence Tomography

Another widespread imaging technique used to detect atherosclerosis is optical coherence tomography (OCT). OCT is an interferometric technique with high spatial resolution and fast image acquisition, capable of providing 3D imaging in vivo. It is widely used in medicine, specifically in dermatology [64], ophthalmology [65], and cardiology [66]. Particularly, in intravascular OCT, a catheter comprising an optical fiber transmits the light and measures the “echoes” of light backscattered by the sample to build an image while it is rotated and pulled across the length of the assessed artery [67]. Therefore, it can distinguish atherosclerotic plaque structures based on grey-scale images.

Previous studies already demonstrated the association between OCT imaging signals and the corresponding atherosclerotic tissues [68,69]: for example, lipid-rich plaques show diffuse borders with signal-poor regions, while fibrous plaques show homogeneous and signal-rich regions, meaning that lipid-rich plaques have higher attenuation and backscattering coefficients than fibrocalcific and fibrous plaques [69]. For this reason, including adequate structural and molecular sources of contrast into the biomodel is crucial, so the OCT phantoms can accurately mimic realistic pathologic conditions [70].

One of the first works validating the utilization of OCT in the prediction of atherosclerotic plaque vulnerability was accomplished by Fleming and co-workers [71]. They have prepared various phantom samples, differing in their composition, and have placed them into quartz cuvettes for imaging. Cholesterol, collagen, calcium, glycerol trioleate, and water were used to produce mixtures approximately similar to the chemical compositions of plaques. A combined spectral and attenuation model was applied to predict the presence of specific molecules accurately [71]. With this cuvette approach, authors could evaluate the contribution of each component in resulting signals. They further expanded the study via exploring a more realistic condition, in which the fat emulsion was underneath a scattering medium. To do so, mayonnaise was injected into the tunica media of fresh, healthy swine aorta. Mayonnaise was chosen due to its favorable composition and scattering signature [71]. Results indicated that the proposed imaging model was able to detect the presence of lipids below varying depths of material tissue, which demonstrates the utility of the system [71].

Although OCT has numerous advantages, it also has some drawbacks that should be corrected. For instance, OCT imaging could mischaracterize lipid-rich plaques due to intrinsic artefacts, so Nam’s team [72] tried to optimize OCT protocol through complementing it with spectroscopic metrics. The proposed method was developed to solve ambiguity problems occurring during the characterization of grey-scale OCT images. Lipid phantom models were developed to validate the OCT system. The authors have prepared three phantoms with different concentrations (28.6%, 16.7%, 9.1%) of lipid-mimicking plaque. Due to its composition and backscattering properties, mayonnaise was chosen to mimic the lipid composition of atherosclerotic plaque. Prepared solutions were filled into a fluorinated ethylene propylene tube to mimic vessel luminal structure [72]. This approach allowed one to quantify the amount of lipid presented on atherosclerotic plaques by means of the spectroscopic OCT method.

Meanwhile, to assess the feasibility of combining OCT and photoacoustic tomography in the detection of the composition and structure of lipid core and fibrous cap, Shang et al. (2017) [73] built a multi-component plaque phantom. Fibrous cap was simulated through mixing gelatin, agar, and collagen, while a combination of cholesterol, phospholipids, and triglyceride mimicked the lipid core. Results revealed that this dual-mode imaging modality was viable in plaque detection in advance of in vivo experimentation, being “a prospect in understanding the effect of structural properties on plaque progression” [73].

### 3.2. Hemodynamic Studies (Flow Models)

Besides the research on optimizing diagnostic tools to assess atherosclerosis development, another growing research interest is investigating atherosclerotic lesion formation as a response to hemodynamic changes. This study is often conducted using flow models reviewed by implementing phantom models [56]. In this context, and due to its transparency, inertia, non-toxicity, optical transparency, and tunable elasticity [74], the silicone rubber polydimethylsiloxane (PDMS) is the preferred material for the replication of vascular walls in hemodynamic studies [75,76,77,78,79]. Moreover, since it has good cytocompatibility, PDMS is commonly applied to in vitro studies, in which cellular adhesion is crucial for an effective cell culture.

**Figure 5:**
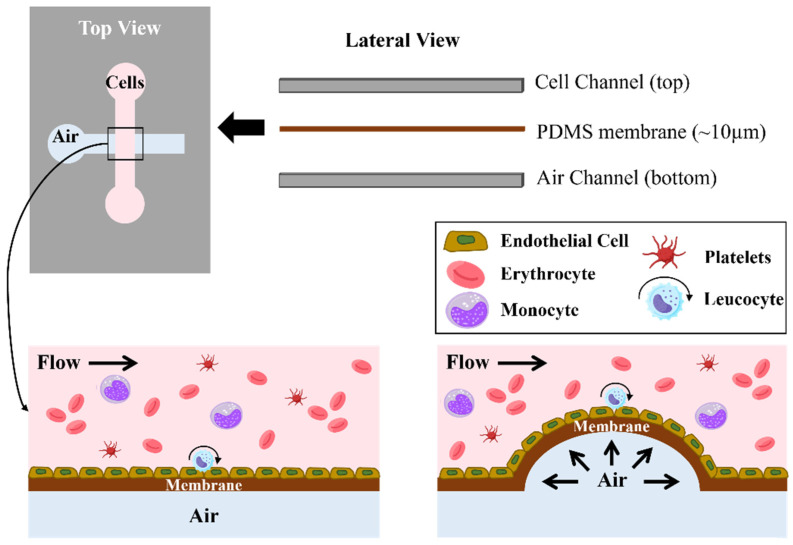
Schematic illustration of the multi-layered microfluidic device, showing the mechanism to produce the 3D stenosis from air pumping. A cell culture channel (**top**) and an orthogonal air channel (**bottom**) are separated by a thin PDMS membrane. When air is pumped into the bottom channel, the PDMS membrane deflects upward, creating a controllable stenosis in the top fluidic channel. (Diagrams and schemes were adapted from [80]).

Although in vitro models are widely used for hemodynamic investigations, they do not fully recapitulate vascular geometries or complex blood flow profiles. Therefore, some researchers introduced a “pneumatically actuated 3D stenosis blood vessel model to study hemodynamics and leukocyte-endothelial interactions using microfluidics” [80]. The multilayered PDMS-based microfluidic device comprised a cell culture channel (top), into which healthy and inflamed blood was perfused, and an orthogonally placed air channel (bottom), separated by a thin PDMS membrane that deflates upwards when the air is pumped into the air channel, creating a controllable 3D stenosis (Figure 5). PDMS was used to improve the adherence of the endothelial cell’s monolayer. Results indicated that, for 80% stenosis, the density of leucocyte adhesion strongly depends on the inflammation degree (varied quantities of tumor necrosis factor-α–TNF-α).

Additionally, flow and shear stress profiles were varied, and distinct leucocyte adhesion patterns were found. Since the authors could discriminate healthy and inflamed blood samples in a dose-dependent manner based on leucocyte adhesion [80], they concluded that leucocyte-endothelial adhesion could be a functional biomarker to assess the risk of atherosclerosis development. Although this work does not replicate atherosclerotic plaque composition, it contributes to the study of the effect of blood flow and composition on the inflammatory response, i.e., atherosclerosis development.

Another team of researchers [81] took a step further and obtained patient-specific flexible phantom arteries using the sacrificial mold method. The mold was 3D-printed using a water-soluble resin, then cast with PDMS and removed by dissolution in water. SLA technology was chosen over the FFF due to its capability of achieving better surface properties and resolution. Flexible and transparent channel networks were imaged, and comparisons with patient data showed good agreement. Results indicated that these biomodels have adequate elastic properties to mimic in vivo fluid-to-wall interactions, validate numerical simulations, and experimentally determine pulse wave propagation. The latter is particularly relevant to estimate vascular stiffness in atherosclerotic vessels indirectly [81].

Nevertheless, other materials can be used to mimic arterial walls. One example is the silicone elastomer Slygard^®^, implemented by Kefayati and coworkers [82], to assess the transitional flow patterns in normal and stenosed carotid artery bifurcation models. Authors used particle image velocimetry to measure flow velocities with high temporal and spatial resolutions. Results confirmed that the flow pattern becomes more turbulent as the stenotic degree increases, instigating higher thrombogenicity [82].

The 3D-printed biomodels have also been used to assess the impact of hemodynamic conditions (blood pressure and flow rate) on the measurement of fractional flow reserve (FFR) [83], which determines the physiological significance of a stenotic lesion. Therefore, researchers [83] have fabricated vascular models with five different stenotic degrees varying from 30% to 70%. VeroClear^®^ rigid material was used in a Polyjet printer to simulate vessel walls and produce tubular structures, in which a range of physiological pressure rates was applied using a flow pump. For a given stenosis percentage, as the aortic pressure increases (characteristic of a myocardial infarction condition), the corresponding FRR value decreases [83]. This work proves the relevance of physical biomodels in investigating the influence of hemodynamic parameters on the measurement of stenosis significance.

Concomitantly, and to investigate the effect of flow conditions across patients, work was developed [84] to create patient-specific 3D printing models using SLA to assess severe aortic stenosis. Calcified anatomic regions were printed with a rigid material, VeroWhitePlus^®^, while soft tissue structures, including the ascending aorta, were replicated with a rubber-like material (TangoPlus^®^). Each model was then coated with a thin layer of silicone [83]. These biomodels demonstrated that, despite the measurement method, the determined aortic valve area depends on the patient-specific transvalvular flow rate [84]. Careful medical analysis should, therefore, be conducted.

Conjointly, these works proved the relevance of biomimicking atherosclerotic disease to predict hemodynamical performances of blood vessels, thus allowing the prediction of the best suitable treatment strategy without using in vivo experimentation and reducing operational stress.

### 3.3. Validation of Numerical Studies

Due to their geometric tunability, numerical tests are often preferred over in vivo experimentation, constituting auxiliary tools to investigate diseases such as atherosclerosis. Typically, numerical studies can assess structural behavior in arterial wall deformation. Studying the blood fluid dynamics is also possible, with computational fluid dynamics (CFD) being the most common method. Furthermore, to study the reciprocal relationship between blood flow and artery deformation, the fluid–structure interaction (FSI) approach is the most adequate [85].

Numerical results must be validated to ensure accuracy despite reproducibility and low expensiveness. The design of biomimicking physical models to accomplish this demanded validation also contributes to the confirmation and complementation of in vitro and in vivo trials.

In an introductory hemodynamic study, researchers have 3D-printed patient-specific models of coronary arteries from CTA images via SLA technology [75]. By implementing CFD, they studied fluid flow profiles and shear rate distributions on the produced 3D vessel geometries and the original CTA-scale 3D vessel geometries. To fabricate the physical biomodels, molds with the intended geometry were fabricated using PIC100 resin, and PDMS was poured into the molds to mimic the vessel wall. CFD was then performed on both vascular geometries (miniaturized designed model and original CTA-scale artery). Comparisons between geometries revealed minor differences in shear rate distributions and no significant differences in fluid flow profiles [75]. The authors concluded that the miniaturization methodology can accurately replicate the CTA-segmented model, and CFD could serve as an auxiliary tool to study stenosis hemodynamics.

**Figure 6:**
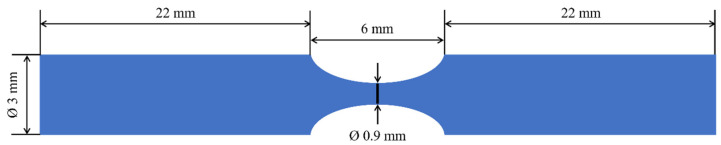
Two-dimensional view of the dimensions of 3D-printed tubular models with a 70% stenosis. Adapted from [23].

Researchers [23] have further studied the influence of model surface roughness on hemodynamical characterization to investigate the correlation between numerical and experimental results. The authors 3D-printed biomodels (via SLA technology using a rigid material) with varying printing resolutions to obtain different surface roughness values. Stenosis was accomplished via creating constrictions in the CAD models (Figure 6). Flow characterization was conducted numerically and experimentally using CFD simulations and high-speed video microscopy techniques, respectively. Results demonstrated the strong dependence of flow measurement on surface roughness. Indeed, comparisons between experimental and numerical results revealed that the most congruent flow visualization profiles appeared for the less-rough surface [23]. This study has disclosed some weaknesses of numerical fluid simulations, namely that they do not consider the microtopography of vascular models, which is certainly presented in the in vivo models. Therefore, attentive assumptions must be carefully conducted until improvements in this field are achieved.

Nevertheless, and as aforementioned, computational simulations can also be implemented to predict the structural behavior of vascular models. The architectural change resulting from stenosis could be significant in assessing atherosclerotic plaque vulnerability. Notwithstanding, structural numerical results should be validated experimentally.

Within this framework, Guarnera and co-workers [86] have proposed a novel investigation based on 3D printing to validate numerical models for biomechanical simulations. The study aimed to obtain the most appropriate association between the in vivo and the in silico processes. For that, they chose materials with mechanical properties similar to those of mimicking tissues and compared the mechanical responses of physical models (replicating in vivo pathological conditions) and the corresponding computational models. Using Polyjet technology, the authors obtained five digital materials with nonlinear mechanical properties through jetting two soft elastomeric materials (VeroClear^®^ and Agilus 30^®^) in different percentages. The resulting materials presented Shore hardness of 30A, 40A, 50A, 70A, and 95A and were used to mimic the lipid pool, tunica media, fibrous cap, fibrotic media, and calcified plaque, respectively. Digital image correlation was implemented to measure the displacements and deformations occurring in fabricated biomodels when inflated with an 800-mmHg pressure balloon. Comparisons between the experimental and the computational results of three numerical methods indicated that the 3D nonlinear finite element (FE) model is the most accurate for predicting the overall behavior of biostructures [86].

These studies highlight the importance of developing physical models to either validate or discard the implementation of numerical methods in predicting mechanical and hemodynamic behaviors of atherosclerotic pathophysiological environments. They also show that research opportunities in this field still exist.

### 3.4. Clinical Practice Assistance

One of the most valuable applications of biomimicking models is related to clinical practice contexts [87,88,89]. Due to its ability to fabricate personalized models without molds, additive manufacturing is commonly implemented to construct patient-specific models capable of accurately replicating the anatomy and pathology of human structures [27]. As already stated [90], cardiovascular 3D-printed biomodels could be used to (1) assist the pre-surgical planning of complex cardiac surgeries, (2) simulate surgical or interventional radiology procedures, and (3) improve doctor-to-patient communication. Successful results have already proven the relevance of such 3D-printed biomodels in the cardiovascular community [91,92,93,94,95,96,97].

Combining high-spatial-resolution imaging techniques with powerful processing software and advanced manufacturing technologies introduces new capabilities in hospital centers [88]. Patient-specific models of cardiovascular pathologies offer complementary information about patients selected for cardiovascular interventions, allowing the revision or improvement of surgical strategies and thus increasing the success rate of these procedures [98].

Flexible soft materials are the favorite to mimic vascular walls when SLA [93,94], digital light processing (DLP) [96], and Polyjet [93,95,99,100,101] technologies are used. On the other hand, due to the low availability of soft filaments, flexible filaments, such as TPU and Filaflex, are preferred [91,93,102] in studies using FFF technology.

There is still work to be conducted regarding mimicking atherosclerotic plaques in clinical practice. Few studies have addressed using different materials to accomplish atherosclerotic vessels [96]. Curiously, 3D-printed stenotic models are mainly achieved through geometrical constrictions created in computer designs and then 3D-printed in just one material.

### 3.5. Assessment of Novel Therapeutic Strategies

Biomodels mimicking atherosclerotic blood vessels can also be utilized to investigate the effectiveness of new interventional techniques, preceding in vivo and in vitro experimentation, especially when they are in the initial validation stages.

For instance, an arterial atherosclerotic plaque phantom was developed to explore the potential of high-intensity focused ultrasound (HIFU) in the treatment of atherosclerotic plaques [103]. This model comprised a biomimicking multicomponent plaque and a 3D-printed tube combining thermoplastic polyurethane (TPU) and copolyester (CPE) to mimic arterial walls. Agar, gypsum, and butter were used to mimic, respectively, fibrous tissue, calcium, and the lipid core and, thus, produce a plaque phantom that was poured into the 3D arterial mimic tube. The amount of plaque removal via the HIFU transducer (4 MHz) was evaluated visually using an X-ray system. A total of 27.1% of plaque phantom was destroyed using the small flat transducer in a slight period (30 s), which has validated the usefulness of this therapeutic technique in future clinical trials [103].

Another approach was carried out [104] to study the feasibility of drilling calcified plaque (DCP) to remove calcified plaques inside arteries. To validate this methodology, they have developed a phantom model consisting of an artery and a calcified plaque. A PVC tube was used to represent the artery, and a bovine cube bone was implemented on the tube wall to simulate calcified plaque. Phantoms have effectively validated the maximum temperature calculated via computer simulation, establishing the diameter and material of the drill bit as the main crucial factors in thermal damage. Therefore, the potentiality of DCP in atherosclerosis treatment was confirmed [104].

Biomimicking models also benefit the study of pharmacokinetics and pharmacodynamics of drugs to treat atherosclerosis. Therefore, researchers [25] hypothesized an artificial atherosclerotic plaque to investigate the influence of plaque composition on drug transport in the arterial wall when a drug-eluting stent is implemented. A hydrogel made of gelatin and alginate was used as a substrate to produce lipid-rich plaque (type IV lesion). Then, liposomes comprising cholesterol, cholesterol palmitate, cholesterol oleate, cholesterol linoleate, and phospholipids were dispersed into gelatin/alginate solutions to obtain a lesion with a lipid core with distinct solid deposits. The results showed that lipids in artificial plaques are a meaningful barrier to drug release. The results have reinforced the importance of atherosclerotic models with a predictable and reproducible location and composition in studying novel treatment options [25].

Regarding the development of new cardiovascular devices, creating anatomically accurate patient-specific models could provide insights into how the device will deform or alter the anatomic configuration or vice-versa [98].

## 4. Conclusions and Prospects

The atherosclerotic disease has become one of the most severe contemporaneous problems, with solid evidence of morbidity and mortality. Many efforts are being made to overturn the catastrophic projections. This has been accomplished via adjusting the preventive measures on healthcare systems and divulging consciousness campaigns, or by improving and designing new treatment and diagnostic strategies. Understanding the complex and controversial process of atherosclerotic pathophysiology is also a goal of research centers to achieve more effective therapeutic approaches. Therefore, biomimicking models fully recapitulating the pathophysiologic conditions of atherosclerotic blood vessels are excellent auxiliary tools to improve the knowledge of atherosclerosis development. These models can validate and optimize cardiovascular devices, imaging systems, and treatment protocols by considering both the hemodynamical and biomechanical phenomena. Moreover, due to their controllability, accessibility, and availability, these biomodels can be very helpful and advantageous in pre-clinical trials, overcoming some limitations of in vivo experimentation, namely the expensiveness, low reproducibility, variability, and ethical issues. These are particularly relevant in the initial stages of the approval process because they provide additional perspectives about the potentiality of a specific protocol or device before the unnecessary use of animals.

Due to the broadness of possible applications of these biomodels, there are, consequently, several crucial properties to be considered in selecting tissue-mimicking materials. Therefore, this review provided a concise overview of materials and processes used to fabricate atherosclerotic vessel biomodels, depending on their final purpose. For instance, optical properties were shown to be the most important when it is intended to optimize imaging systems. On the other hand, in flow studies, chemical properties must be replicated so the fluid behaves physiologically. Mechanical properties, however, are always considered regardless of the intended application. This is translated into the number of studies using soft, flexible materials to mimic arterial walls and more rigid materials to mimic atherosclerotic plaques.

Nonetheless, scientific gaps in biomodel creation and materials design still need to be filled, ideally gathering all the biological properties. By accomplishing this, it will be possible to improve the understanding of atherosclerotic disease and, thus, develop therapeutic and diagnostic tools much more efficiently.

## Figures and Tables

**Table 1:** Atherosclerotic plaque classification according to the American Heart Association [1,28].

Lesion Type	Nomenclature	Lesion Characteristics–Main Histology Properties
Type I	Early lesion	Initial lesion with foam cells
Type II	Fatty streak	Fatty streak with multiple foam cell layers
Type III	Pre-atheroma	Pre-atheroma with extracellular lipid pools
Type IV	Atheroma	Atheroma with a confluent extracellular lipid core
Type V	Fibro-atheroma	Fibrotic and calcified layers with lipid cores
Type VI	Rupture lesion	Complex plaque with possible surface defect
Type VII	Calcified lesion	Calcified plaque
Type VIII	Fibrotic lesion	Fibrotic plaque without lipid core

## Data Availability

Not applicable.
